# A case study of suicidality presenting as a restricted interest in autism Spectrum disorder

**DOI:** 10.1186/s12888-019-2122-7

**Published:** 2019-04-27

**Authors:** Luisa Weiner, Amandine Flin, Jean-Baptiste Causin, Sébastien Weibel, Gilles Bertschy

**Affiliations:** 1 Department of Psychiatry and Addictology, University Hospital of Strasbourg, Strasbourg, France; 2 INSERM 1114, Department of Psychiatry, 1 place de l’hôpital, 67000 Strasbourg, France

**Keywords:** Autism spectrum disorder, Suicide, Suicidal thoughts, Restricted interest, Executive dysfunction, Case study

## Abstract

**Background:**

Suicidality has been under-researched in autism spectrum disorders (ASD). Most studies have linked increased suicidality in ASD to psychiatric comorbidities such as depression. Here we investigated, from a neuropsychological and clinical standpoint, the relationship between core ASD symptoms, i.e., restricted behaviors and social and communication impairments, and the suicidal behaviors in an adult male individual with ASD, with no psychiatric comorbidities.

**Case presentation:**

We report the case of a 21-year-old male with ASD who attempted suicide twice, in the absence of other psychiatric diagnoses. His behavior and communication skills were rigid. His suicidality was characterized by a rigid, detailed, and pervasive thinking pattern, akin to restricted interests. Consistently, from a neuropsychological standpoint, we found below-average planning and attention skills, and mind-reading skills were rigid and lacked spontaneity.

**Conclusions:**

Our case-study suggests that specific clinical and neuropsychological dimensions might be related to suicidal behaviors in ASD. Clinically, the repetitive and rigid suicide-oriented thinking of our patient was not part of a depressive episode. Instead, it followed a purely logical, inflexible, and pervasive reasoning pattern focused on a topic that fascinated him – i.e., suicide --, akin to restricted behaviors. From a neuropsychological standpoint, restrictive suicide-oriented thinking in our patient seems to be related to attention and executive anomalies that have been linked to repetitive and restricted behaviors in ASD. New tools need to be developed to assess persistent suicidal thoughts in this population, as they might be related to intrinsic features of ASD.

## Background

Autism Spectrum Disorder (ASD) is a neurodevelopmental condition characterized by two clusters of symptoms: i.e., impaired social communication and interactions, and restricted and stereotyped behaviors [1].

A growing number of studies has shown that the prevalence of psychiatric comorbidities in adults with ASD is high [2], but only a few studies have focused on the risk of suicide in this population. The prevalence of attempted suicide in ASD has been reported to be higher than in the general population [3, 4], but also higher than the prevalence reported in schizophrenia [5] or in depression [6]. Indeed, the rates of attempted suicide among individuals with ASD varies between 7 and 47% [7], and 66% of adults with ASD already have experienced suicidal thoughts [5]. Individuals with ASD constitute between 7.3 and 15% of the entire population presenting with suicidal behaviors [8] despite representing only 1% of the general population [9].

Although some studies have suggested that ASD is independently associated with increased risk of suicide [3], due to some of its core symptoms – e.g., impaired communication abilities and social isolation [10] --, others have reported that this risk is only increased in the case of psychiatric comorbidities such as mood disorders [5, 10–13], anxiety disorders [11] or psychotic disorders [14]. Risk of suicide in individuals with ASD is also increased in the case of intellectual giftedness [4, 13, 15, 16], self-harming behaviors [7], family history of suicidality [14, 17], experience of violence, harassment or victimization [8, 15–17], and low self-esteem [18]. Other risk factors include male gender [7], belonging to an ethnic minority or having a lower socio-economic background [8].

In this paper we present a detailed clinical and neurocognitive description of a 21-year-old male patient admitted in an inpatient psychiatric unit for attempting suicide. No diagnosis had been established prior to his hospitalization, although it was his second suicide attempt. ASD diagnosis was established during the hospitalization. This suicide attempt occurred in the absence of a depressive episode or any other psychiatric disorder, raising the hypothesis of a specific link between his suicidal behavior and the core characteristics of ASD. More specifically, we will explore the link between socio-communicational impairments and restricted ideas and the emergence and persistence of suicidal thoughts. From a neuropsychological standpoint, executive dysfunctions, such as cognitive rigidity and poor decision-making skills, may constitute a vulnerability trait for the risk of suicide [19, 20]. Deficient mindreading and emotion recognition skills have also been found to be related to suicide attempts [21]. Both executive and social cognition dysfunctions have been reported in a large number of studies in children [22, 23] and adults with ASD [24, 25]. We will investigate here whether the persistent suicidal ideation in this patient might be associated with cognitive rigidity, restricted interests, and socio-communicational impairments, assessed both clinically and via neuropsychological tasks.

## Case presentation

Mr. A is a 21-year-old single man, who lives with his mother. He was admitted to our inpatient psychiatric unit in 2018, following a suicide attempt by hanging. His suicide attempt was the result of a fine-grained “logical analysis”, which he described to several professionals in a detached and didactic tone, detailing it step by step. He saw no point in “living his life until the end”, comparing it to a movie whose first scenes “did not thrill him”. He also gave a similarly meticulous, unemotional breakdown of his reasoning in the presence of his mother. His suicidal thoughts were not part of a depressive episode: no reduced activity, low energy or mood or anhedonia were reported. On the eve of the suicide attempt, the patient went to school, and performed his usual daily activities, including his hobbies (e.g., playing the piano). He saw his psychiatrist and his mental status was stable. His score on the *Beck Depression Inventory* (BDI) [26] was in the normal range (3/63): he reported only a slight loss of appetite (1/3), along with suicidal thoughts (2/3). No trigger for his hanging attempt was identified. During the dinner before said attempt, he had a dispassionate philosophical discussion with his mother on the topic of suicide, in which he advocated his right to end his life. He reports that he had not yet decided to attempt suicide by then, but thought it was his responsibility to prepare his mother to the possibility. The attempt failed due to a mechanical problem with the rope and the hanging point. He then quietly went to bed and fell asleep. However, he forgot to switch off the bathroom light and to remove the rope, leading his mother to find out what had happened during the night.

Mr. A. reported that his suicidal thoughts started when he was 18, following an unrequited infatuation with a classmate – the result of a rational decision: he had decided to “fall in love” with her. His love remained unrequited and the girl had a short romantic relationship with a friend Mr. A. had introduced to her. While he did not mind the relationship per se, upon learning that it was short-lived, and thus just “for fun”, Mr. A. became upset. He started having “obsessive negative thoughts”, and attempted suicide by jumping from a window. He survived and was hospitalized for 8 months, first at an intensive care unit, then at a rehabilitation unit. His outpatient psychiatric follow-up began at that time. No psychotropic drugs were prescribed to him. However, his suicidal thoughts lingered, characterized by a restrictive, rigid pattern, which was not associated with a depressive mood.

When we met him, Mr. A. was a first-year engineering-student in the south of France. He liked school and said he had a few friends, but his relationships with his peers lacked spontaneity – i.e., he had to study their behavior in order to know “what to say”. When prompted, he told us he felt his emotions were “overwhelming”, and that he was unable to regulate them. There was a discrepancy between the content of his speech and his facial expressions, which were neutral. His communication skills were qualitatively rigid, as it was difficult for him to grasp the implicit meaning of a number of phrases. Many open-ended questions had to be rephrased, as he considered them “too vague”. His speech was monotone, but his vocabulary was rich, precise, and displayed no morphological abnormalities. He exhibited an adherence to non-functional routines; for example, he always took his shower in the exact same way, and studied on the same table since childhood. Mr. A. presented with motor stereotypies, such as rocking back and forth when he felt anxious or pensive. Sensory abnormalities were also reported concerning his hearing, temperature perception and nociception. For example, Mr. A. has never reacted to painful stimuli, and wears the same clothing irrespective of the weather. Restricted and all-consuming interests were also reported: as a child, Mr. A. showed periodical interest in topics such as Ancient Egypt and Middle-Age History, which became his sole topics of discussion. When we met him, his spontaneous communication was limited. Mr. A. explained that he disliked chatting, as he did not know what to say. His discussion topics were limited to his areas of interest (i.e., computer science), but he could also easily talk about his suicidal thoughts. His developmental history, as well the current clinical picture, were consistent with ASD diagnosis according to the DSM-5. ADI-R [27] scores were also consistent with the diagnosis, as results were above the cut-off in the three ASD core domains of social interactions (score 10; cut-off ≥10), communication (score 9; cut-off ≥8) and behaviors (score 4; cut-off ≥3).

A comprehensive neuropsychological battery was administered. Mr. A.’s scores on the *Wechsler Adult Intelligence Scale -Forth Edition* (WAIS-IV) [28] indicated that he had an above-average IQ (FSIQ = 115), with very high verbal abilities (VCI = 128), whereas his perceptive reasoning and working memory abilities were 1 standard deviation above average (POI = 114; WMI = 112, respectively). Processing speed (PSI = 89) and selective attention were the main weaknesses in his functioning (circa 1 SD below-average). Sustained and divided attention performance were average on the Test for Attentional Performance [29]. Set-shifting, verbal initiation and inhibition abilities were above average, as were verbal and spatial working memory abilities. Planning was efficient but required much time, as assessed by the Tower of London [30]; verbal and non-verbal long-term learning were also efficient. However, peculiarities were observed in the California Verbal Learning Test [31], as he learned the word list in order of appearance, instead of clustering words in a semantic fashion. Furthermore, his learning curve was irregular, owing to attention and executive difficulties as well as to idiosyncratic learning strategies. His copying style of the Rey-Osterrieth Complex Fig. [32] was detail-based: he drew a small part of its outline before including details into the frame. Performance on the Reading the Mind in the Eyes Test [33], assessing facial emotion recognition, was particularly slow, hence not spontaneous. Mindreading skills were rigid in the faux-pas task [34], with the same sentences repeatedly produced to describe others’ mental states. Overall, our results are suggestive of attentional and processing speed difficulties; planning skills and facial emotion recognition are efficient, but costly and lack spontaneity; information processing is concrete and detail-based; mentalization abilities are rigid.

## Discussion

We reported here a case study which illustrates the relationship between increased suicidality in a young man recently diagnosed with ASD, and some of the core clinical and neuropsychological features of ASD. Importantly, unlike previous studies, e.g., [5, 10, 11], our patient did not present with any other psychiatric diagnoses, such as depression. Our case study thus adds to results recently reported by two three studies suggesting that ASD, especially in high functioning individuals, might be independently associated with an increased risk for suicide [3, 4, 35].

The absence of psychiatric comorbidities raises the question of whether the persistence of suicidal thoughts was associated with ASD-related features, which had not been diagnosed prior to his second suicide attempt. Both clinically and neuropsychologically, our patient presented with features typically found in individuals with high functioning ASD. Aside from executive and social cognition abnormalities, he presented with a detailed-focused information processing style, and a heterogenous intellectual functioning, with very high verbal abilities, whereas processing speed was slow, which has been described in other studies with individuals with high functioning ASD [36].

Social and communication impairment has been previously reported to be a risk factor for suicide attempts in ASD [10]. In our patient, social and communication impairment was involved in his inability to seek help, and to identify, communicate and regulate his own emotions – which he described as “overwhelming”. Emotion dysregulation in adults with ASD is an understated clinical feature, which has been associated with non-suicidal self-injury [37], but, to our knowledge, studies focusing on the relationship between emotion dysregulation and suicidality in ASD are lacking.

From a neuropsychological standpoint, his emotion and mind-reading skills lacked spontaneity, reflected by the abnormally long time required to complete the tasks. The rigidity of his suicidal thoughts may be linked to the lack of behavioral flexibility observed here both clinically and neuropsychologically. His suicidality was characterized by a rigid, detailed, and pervasive thinking pattern, akin to restricted interests and behaviors. Moreover, our patient was unable to fully grasp the impact of his behavior on others, such as his mother, and the descriptions of his suicidal thoughts followed the same pattern regardless of the context. Past studies have provided evidence for the relationship between executive dysfunction, i.e., cognitive rigidity, and restricted and repetitive symptoms in ASD (e.g., [38]). Our case study is consistent with such findings, as his suicidal thoughts were particularly repetitive, hence rigid and restricted, in nature. Recently, Unruh et al. [39] have found that repetitive negative-oriented cognition is a core feature of ASD, which constitutes a link between the disorder and the risk for depression. In our case study, the repetitive and rigid suicide-oriented thinking of our patient was not part of a depressive episode. Instead, it followed a purely logical, inflexible, and pervasive reasoning pattern focused on a topic that fascinated him – i.e., suicide. His suicidal thoughts remained present despite numerous psychotherapy sessions. Like restricted interests and behaviors in ASD [40], his suicidal thoughts were particularly treatment-resistant.

It is important to note that our patient had not been diagnosed with ASD prior to his second suicide attempt, and this might have contributed to the severity of both attempts. Kato et al. [6] found that individuals with ASD who attempted suicide (i) had persistent rather than specific stressors, (ii) used more lethal means, and (iii) were less connected to psychiatric services. Late diagnosis in our case might have contributed to the persistence of stressors related to undiagnosed ASD in everyday functioning, on the one hand, but also to the lethal means used in both attempts; even though by the time of the second attempt he had a psychiatric follow-up, no diagnosis had been established. During the disclosure interview, suicidality and emotion dysregulation were explained within the framework of the ASD diagnosis; akin to findings reported in past studies (e.g., [41]), ASD diagnosis felt as a “relief”, according to the patient. Although insight into ASD-related difficulties remained poor, probably because of cognitive inflexibility [42], diagnostic disclosure increased hope in terms of support that could be offered for his communication and emotion regulation difficulties. Suicidal ideation lingered, but was no longer as pervasive, owing to the fact that his thoughts were also focused on other topics (i.e., on his studies and hobbies).

Suicidality in people with ASD remains poorly understood, and under-researched. Our case-study provides evidence for the involvement of specific clinical and neuropsychological dimensions as risk factors for suicidal behaviors in ASD. Because ours is a case study, we cannot ascertain the relationship between our cognitive and clinical findings, specifically suicidal ideation. Studies are needed to empirically address the relationship between suicidality and neurocognitive traits of ASD.

Moreover, new tools need to be developed to assess and offer support to individuals with ASD presenting with persistent suicidal thoughts, as they might be related to intrinsic features of ASD. This is crucial as suicidality is particularly difficult to assess in ASD, given their peculiar clinical presentations, and the absence of early-warning signs due to social and communication impairment [8].

## Abbreviations

ADI-R: Autism Diagnostic Interview-Revised
ASD: Autism Spectrum Disorders
BDI: Beck Depression Inventory
DSM-5: Diagnostic and Statistical Manual of Mental Disorders-Fifth Edition
IQ: Intelligence Quotient
SD: Standard Deviation
WAIS-IV: Wechsler Adult Intelligence Scale -Forth Edition
